# Form-Stable Phase Change Material with Wood-Based Materials as Support

**DOI:** 10.3390/polym15040942

**Published:** 2023-02-14

**Authors:** Farzana Hanif, Muhammad Imran, Yuang Zhang, Zhaoying Jia, Xiaohe Lu, Rongwen Lu, Bingtao Tang

**Affiliations:** State Key Laboratory of Fine Chemicals, Frontier Science Center for Smart Materials, School of Chemical Engineering, Dalian University of Technology, Dalian 116024, China

**Keywords:** heat transfer, leakage proof, scanning electron microscope, shape-stabilized phase change materials, thermal energy storage, wood

## Abstract

Building shape-stable phase change materials (PCMs) are crucial for their practical applications. Particularly, it is vital to utilize renewable/recyclable biomass media as the support material of form-stable PCMs. In this review article, we summarized the recent developments for building form-stable PCMs consisting of wood as a supporting material, either carbonized wood or wood composites. Moreover, the electrothermal conversion and photothermal conversion of form-stable PCMs based on carbonized wood are also demonstrated. In addition, the current technical problems and future research developments of wood-based PCMs are discussed, especially the leakage problem of PCMs during the phase change transition process. All of this information will be helpful for the in-depth understanding and development of new PCMs suitable for wide application perspectives.

## 1. Introduction

In recent years, phase change materials (PCMs) have gained significant popularity and recognition due to their extensive commercial uses, such as for building wallboards [1,2,3,4], bedding and sportswear accessories, food conservation, heat recovery, etc. [5,6,7]. Furthermore, the industrial applications of PCMs also include cold storage applications, automotive, spacecraft, textile, batteries and thermal management systems [8,9]. However, the use of PCMs as thermal energy storage media depends on their nature and bearable temperature ranges [10,11]. Thus, PCMs can be commonly divided into organic compounds, inorganic compounds and metallic PCMs. These PCM classes have unique benefits and drawbacks. For instance, inorganic PCMs frequently exhibited high phase change enthalpy and super cooling behavior, however, they may be corrosive and frequently degrade after repeated heating cycles. Metallic PCMs typically have high heat conductivity, but their applications are restricted by their relatively high melting points. Compared with these PCM materials, organic PCMs showed good thermal stability, self-nucleating properties and non-toxicity behavior [12,13]. Moreover, organic PCMs are non-corrosive, low cost, and have a wide range of available phase change temperatures; they are highly desired PCMs [14]. However, the major drawback of organic PCMs is their inadequately low thermal conductivity, which reduces the rate of heat storage and release. Additionally, the leakage or flammability in most of these PCMs during heat storage is the main barrier [15,16,17].

This review focuses on studying the transmittance of energy storage wood for sunlight transmission and utilization and the leakage problem of phase transition of PCMs. The use of PCMs in buildings, solar water heaters, sustainability and encapsulation techniques have been the subject of numerous studies [18,19,20]. These materials can effectively control indoor temperature and save energy. Numerous benefits can be obtained from these materials, such as shape stability and the ability to provide more convenient water-sensible heat transfer. An appropriate increase in the thermal conductivity of PCM can increase the heat storage and release rate, which is more conducive for temperature regulation. Nevertheless, the relative increase of thermal conductivity is still within the acceptable range of thermal conductivity for wallboard applications.

Practical applications of organic PCMs require a proper encapsulation structure to overcome leakage problems. Encapsulation is a way of casing PCMs with an appropriate covering or outer material to preserve the PCM’s liquid or solid phase and maintain it apart from the fluid substance around it [21]. In this field, chemists have contributed significantly to investigating novel support materials for creating form-stable PCMs [22]. This strategy emphasizes promoting the contact between the liquid phase PCM and the carrier substance in order to prevent liquid phase PCM leakage [23].

Various support materials, i.e., biomass-based materials, can serve as an excellent scaffold for constructing high-performance phase change composites. Especially biomass-based porous support substances with an eco-friendly and renewable nature, like pomelo peel, radish, wood, etc., have drawn a lot of attention [24,25,26,27,28]. Among these, wood has unique properties compared to other materials, and because of its multiple pore sizes, chemical composition and high specific gravity [29], which can provide enough adsorption sites and strong capillary adsorption for organic PCMs to prevent the leakage of fluid. Moreover, the carbonization of wood is easy, and the carbonized wood readily produces porous carbon with high thermal conductivity. To increase heat storage and release rate, a composite form-stable PCM with improved thermal conductivity can be made using wood as a porous support material. Therefore, it is possible to create form-stable PCMs with higher loading capacities using wood, i.e., carbonized wood.

A few review articles on the characteristics and commercialization of various composite PCMs, such as carbon-based PCMs and nanoporous shape-stabilized PCMs, have previously been published [16,30]. However, an overview of form-stable PCMs using wood materials as support has not been presented. Therefore, we attempted to give an overview of the recent developments in creating form-stable PCMs with wood as the supporting material. Moreover, this review will also provide an outline of the methodologies for the construction of wood-based form-stable PCMs used in different applications.

## 2. Issues in PCMs and the Development of Wood-Based Form-Stable PCMs

The fastest-developing techniques for increasing thermal energy utilization efficiency in thermal energy storage and management using PCMs are critical because the imbalance between thermal energy supply and demand has grown to be a serious problem in the current era. However, in order to effectively use thermal energy and prevent the leakage of solid-liquid PCMs, researchers have introduced several natural supporting materials. A porous material is one of the best materials to address PCM leakage issues. As previously mentioned, wood is a naturally porous material with low cost, abundant resources, non-toxic nature and excellent chemical stability. As such, it can more effectively encapsulate PCMs [31,32,33]. The principal mechanism for the synthesis of form-stabilized PCMs with wood is as follows: firstly delignification method is used to increase the porosity of natural woods. In this way, the shape-stabilized phase change composites benefit from the treatment because it not only reduces the weight of the matrix but also gives PCMs a large amount of package space, which helps to increase their ability to store heat. Ultimately, the delignified wood is immersed into the PCMs under vacuum infiltration or direct impregnation methods. Noteworthy to mention here, the cellulosic scaffold derived from wood by partial or full removal of lignin without altering the hierarchically aligned cellulosic structure is called delignified wood.

**Figure 1:**
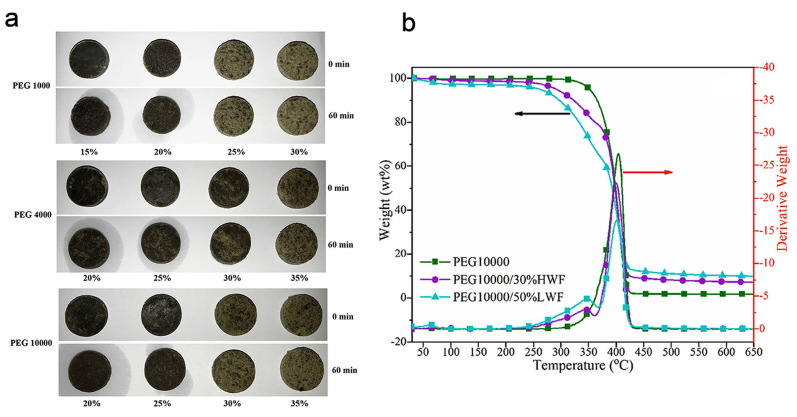
(**a**) Photographs of leakage test; (**b**) thermogravimetric and differential thermogravimetric analysis curves of PEG/HWF shape-stabilized PCMs. HWF stands for high length-to-diameter ratios of wood flour and LWF stands for low length-to-diameter ratios of wood flour. Reproduced with permission from reference [34], copyright 2019, Elsevier B.V.

Yuan et al. used wood flour to impregnate polyethylene glycol (PEG) as PCMs (Figure 1) [34]. Using a straightforward vacuum distillation method, they synthesized and compared various high length-to-diameter ratios of wood flour with low length-to-diameter ratios of wood flour where different molecular weights of PEG were used. By adjusting the mass ratio, the high length-to-diameter ratios of wood flour content were changed in order to examine the impact of high length-to-diameter ratios of wood flour on PCM performance. For instance, to exploit the maximum absorption ratio without causing the melted PEG to leak from the composites, the high length-to-diameter ratios of wood flour mass contents in PEG/high length-to-diameter ratios of wood flour PCMs were set to 15 wt%, 20 wt%, 25 wt%, 30 wt%, and 35 wt%. High length-to-diameter ratio of wood flour porosity study was compared with low length-to-diameter ratios of wood flour. This study demonstrated that the high length-to-diameter ratio of wood flour is more porous than the low length-to-diameter ratios of wood flour and that the high length-to-diameter ratio of wood flour may exhibit stronger PEG adsorption behavior. The microstructure and morphological characteristics of high length-to-diameter ratio of wood flour revealed irregular strip fiber fragments, which suggested that the PEG could fill the porosity of high length-to-diameter ratio of wood flour through surface tension, hydrogen bond interactions, and capillary force. The ability of high length-to-diameter ratio of wood flour and low length-to-diameter ratios of wood flour to absorb and leak at various ratios was tested. In PEG1000/15 wt% high length-to-diameter ratio of wood flour composites (provide less surface area), it showed that the PEG segments quickly melted into a liquid and leaked. In contrast, the PEG segments in PEG1000/25 wt% high length-to-diameter ratio of wood flour PCMs (having large surface area) were able to keep their original shape throughout the heating process without any liquid leakage (Figure 1a).

Although significant PEG content should have a higher wood flour content to prevent leakage and maintain its shape, PEG1000 only needed 35 wt% of low length-to-diameter ratios of wood flour. High length-to-diameter ratio of wood flour had a higher adsorption capacity than low length-to-diameter ratios of wood flour for PEG with the same average molecular weight. This finding could be explained by the wood flour porosity, which has varying length-to-diameter ratios. The low molecular weight PEG (having shorter chain length) and lower viscosity at high temperatures make it more likely to be adsorbed into these wood flour holes through surface tension or capillary force interactions. PEG with lower molecular weight should, however, contain more molar amounts of -OH groups under the same mass fraction, which is more advantageous for the hydrogen bond adsorption of PEG onto wood flour. Therefore, wood flour may more readily absorb PEG with lower molecular weight and provide better anti-leakage performance. According to the chemical composition, there was no chemical reaction between PEG, high length-to-diameter ratios of wood flour/low length-to-diameter ratios of wood flour shape-stabilized PCMs; only a physical interaction took place.

Most PEG segments in composites with low PEG content are either adsorbed on the surface of delignified wood flour or confined within its pores, which prevents them from crystallizing. Because they can be mimicked by accelerating the thermal cycling process in long-term practical applications, thermal stability and reusability are crucial for PCMs. After 100 thermal cycles, there was a small change in the phase transition temperatures, but it had no real impact on the thermal energy storage capabilities. The outcome demonstrated the good chemical stability and thermal dependability of PEG/high length-to-diameter ratios of wood flour shape-stabilized PCMs. These PCMs were scanned in the temperature range of 30–650 °C for thermal stability, PEG lost most of its weight when the temperature was over 500 °C (Figure 1b). However, these samples did not show apparent weight loss when the temperature was <100 °C. We conclude that the photothermal performance of these PCMs showed good thermal reliability and shape/chemical stability with the melting latent heat of 108.6 J g^−1^. Moreover, the PEG as PCM was extensively used with mesoporous carbon (as support) for excellent energy storage capacity and thermal conductivity [35]. It is concluded that the construction of PEG-based PCM is advantageous in terms of shape stability, heat resistance and enhanced thermal conductivity also provides us the flexibility to design shape-stabilized composite PCMs according to the actual requirements suitable for use in thermal energy storage applications.

**Figure 2:**
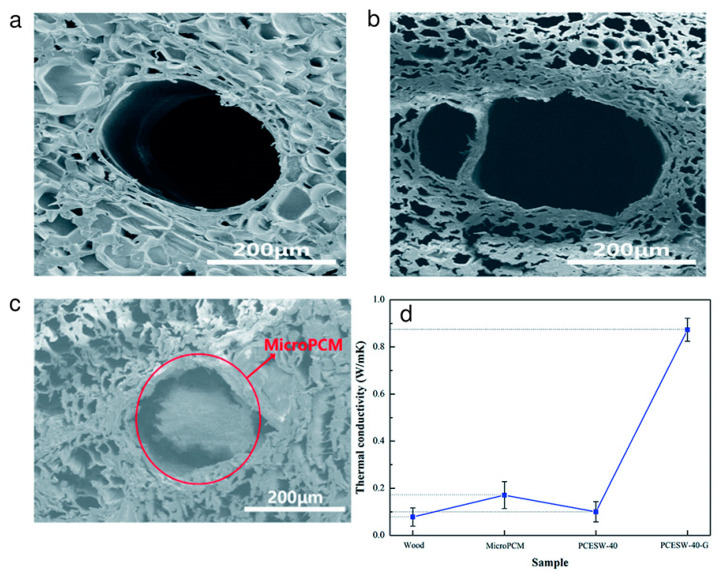
(**a**) Scanning electron microscopic (SEM) images of balsa wood before delignification; (**b**) SEM image of delignified wood; (**c**) microstructure image of phase change energy storage wood and (**d**) thermal conductivity curves of phase change energy storage wood. Adopted from reference [36], copyright 2020, Royal Chemical Society.

Sun et al. combined balsa wood with microencapsulated PCM (microPCM) and graphene (as support) to create the phase change energy storage wood (Figure 2) [36]. This microencapsulation technology is the best way to stop the PCM from leaking or changing volume during the phase transition process, which also increases the PCM surface area for heat transfer [2]. Due to low density and high porosity, balsa wood was chosen to encapsulate the microPCM. With the aid of a mixture solution of NaOH (2.5 mol L^−1^) and Na_2_SO_3_ (0.4 mol L^−1^), the delignification process was carried out. After delignification, the porosity of the delignified wood increased by 18.2%, and the average diameter of the pores enhanced (2.7 to 4.3 mm), which raised the possibility for the incorporation of microPCM (Figure 2a,b). The vacuum impregnation method was used to load the microPCM with an average diameter of 6.5 µm into the delignified wood. Additionally, graphene was included in the impregnation procedure to increase the thermal conductivity of phase change energy storage wood material. Note that graphene is a support material and it is used for enhancing the performance of microPCM. Figure 2c shows the uneven distribution of microPCM found in wood vessels leads to inhomogeneous heat transmission of wood. However, the addition of graphene increased the melting and freezing enthalpies to 44.3 J g^−1^ and 42.5 J g^−1^, respectively, demonstrating that graphene could improve the energy storage ability of phase change energy storage wood material. Other than that, graphene improved the thermal conductivity of phase change energy storage wood (0.873 W m^−1^ K^−1^) and prevented damage to microPCM brought on by inhomogeneous heat transmission (Figure 2d). Moreover, graphene clogged the wood vessels, which prohibited the PCM from leakage and led to the broken microPCM.

**Figure 3:**
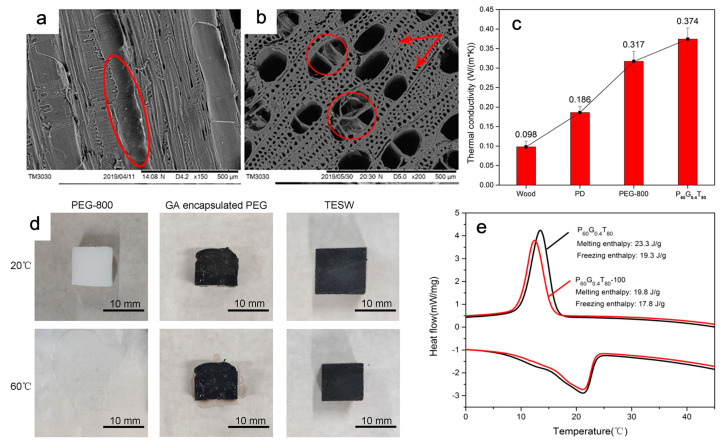
SEM images taken after impregnation of PEG/GA into the wood vessels; (**a**) radial section image (**b**) cross-section image, red circles and arrows in (**a**,**b**) show the successful filling of PEG/GA; (**c**) thermal conductivity performance comparison of wood, PD, PEG and TESW; (**d**) comparison of shape stability of samples and (**e**) cycles of thermal stability of TESW. Note: GA = graphene aerogel, PD = pure wood, PEG = polyethylene glycol, TESW = thermal energy storage wood. Adopted from reference [37], copyright 2020, IOP Publishing.

Later, a thermal energy storage wood using poplar wood as the supportive matrix and graphene aerogel encapsulated PEG as the phase change material to inhibit leakage was reported (Figure 3) [37]. Poplar wood has pores ranging from 10 to 400 μm, identified as natural porous material. On the other side, the particle size of PEG is approximately 1.2 nm, and the size of graphene oxide is <1 μm; these pores of poplar wood offer a high possibility of impregnating the mixture of PEG and graphene oxide. Graphene aerogel is made using the hydrothermal method by combining graphene oxide as the raw material and vitamin C as a reducing agent. PEG was encased in graphene aerogel and used as a PCM. PEG/graphene aerogel was successfully loaded into the vessels after impregnation (Figure 3a,b). The net structure of graphene aerogel was maintained during encapsulation, and it incorporated well with PEG. There was no obvious interface between graphene aerogel and PEG, which was intended to promote good compatibility. Additionally, the cross-linking structure of graphene aerogel and the capillary effect of wood helped to effectively solve the leakage issue caused by melting of PEG. The thermal conductivity increased by about 274% compared with pure wood after the graphene aerogel was added (Figure 3c), reaching 0.374 W m^−1^ K^−1^.

As a result, thermal energy storage wood with high thermal conductivity is advantageous for quick thermal energy exchange to control indoor temperature. Two criteria, such as shape stability and thermal cycling stability, were used to evaluate the viability of thermal energy storage wood. The anisotropic porous structure of the wood and graphene aerogel, which are advantages of the 60 °C heat treatment, prevented leakage in the thermal energy storage wood in comparison to PEG and graphene aerogel encapsulated PEG (Figure 3d). The melting and freezing enthalpies of thermal energy storage wood after 100 thermal cycles were 19.8 J g^−1^ and 17.8 J g^−1^, respectively, showing that thermal energy storage wood has a high degree of stability both in shape and thermally (Figure 3e). In addition, the prepared thermal energy storage wood material exhibited melting and freezing points of 20 °C and 15 °C, respectively, which is suitable for human comfortable temperature range.

**Figure 4:**
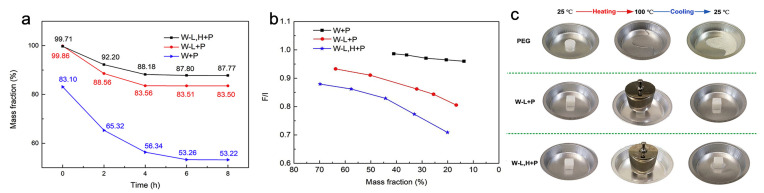
(**a**) Leakage profile data (PEG mass fraction as a function of time) of the balsa wood-based form-stable composite PCMs; (**b**) ratios of experimental phase change enthalpies w.r.t the theoretical phase change enthalpies for various PEG masses loaded in form-stable composite PCMs and (**c**) seepage tests images for pure PEG and wood-derived composite PCMs under external force and longitudinal vascular directions W-L+P and W-L, H+P. Adopted with permission from the reference [38], copyright 2020, Elsevier B.V.

By creating a balsa wood-based 3D framework with PEG, Rojas et al. prepared a form-stable composite PCM for managing thermal energy (Figure 4) [38]. They used balsa wood after careful lignin removal while preserving hemicellulose in the wood. Because completely delignified wood has expanded the porosity and increased to 15% the ratio of encapsulation, it remains vulnerable to leakage when subjected to an external force. Two types of wood samples were observed to make a better comparison; one was delignified with NaClO_2_ for selective lignin removal, and the other sample was treated for up to 6 h with a 10 wt% NaOH solution, decreasing the hemicellulose content after selective delignification. Then, both samples were loaded to PEG through the vacuum impregnation method to prepare form-stable composite PCMs. Removal of lignin and hemicellulose expanded the pits and eliminated the thin wall structure between the fibrous tracheids, improving the permeability of the scaffold. Resultantly, a relatively high degree of encapsulation was observed for the form-stable composite PCMs. Fourier transform infrared spectral results showed the high loading of PEG and hydrogen bond interaction between the PEG scaffolds. It was also suggested that only a physical interaction exists between the PEG and substrate, whereas a chemical interaction may destroy the phase transition performance of the form-stable composite PCMs. Further, the leakage test revealed that lignin and hemicellulose removal enhanced the holding or encapsulation capacity for melted PEG. 

For various samples (Figure 4a,b), the maximum holding time for the residual PEG mass after 8 h was increased from 53.2 to 87.8%. Pure PEG shows phase transition enthalpies of 168.2 J g^−1^ and 158.4 J g^−1^. In comparison, form-stable composite PCMs exhibited enthalpies of 134.3 J g^−1^ and 125.3 J g^−1^, during melting and solidification, respectively, which are almost close values to neat PCM and are desired for wide thermal energy storage. In this work, form-stable composite PCMs were prepared to show outstanding stability together with negligible PCM leakage, even when the ambient temperature is higher than the PCM’s melting point. The distinct hierarchical structure of wood within the cell walls further gives its superior mechanical properties. In this paper, the author used a mechanical compressibility technique on balsa-based scaffolds, both before and after treatment, to test these effects. Native balsa wood did not change in longitudinal directions under the maximum mechanical load; it compressed somewhat after lignin removal and under high compressibility rates but recovered greatly after unloading. Therefore, leakage under external force is anticipated; consequently, to assess the leakage of PEG the samples were observed under 200 g weight with the pressure of 2 kPa during the heating process (Figure 4c). Form-stable composite PCMs maintained their original shape with high mechanical strength and holding capacity and showed no noticeable PEG leakage, even on cooling and unloading. The superiority of this material is that it shows a high latent heat storage capacity of 134 J g^−1^ and low supercooling of 12 °C. In Table 1, we summarize the major physicochemical parameters of the representative PCMs materials discussed in this section.

## 3. Hybrid Composites of Wood, PCMs and Polymer

**Figure 5:**
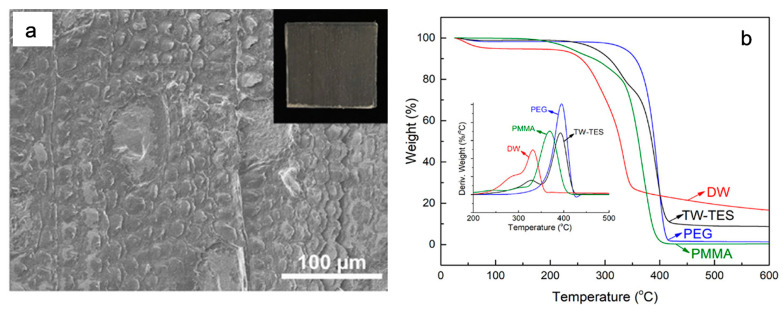
(**a**) SEM micrometre graph showing the successful impregnation of DW with PEG/PMMA; (**b**) the thermogravimetric analysis and differential thermogravimetric analysis curves of DW, neat PEG, neat PMMA and TW-TES composite. Abbreviations are: DW = delignified wood, PMMA = polymethyl methacrylate, and TW-TES = transparent wood for thermal energy storage. Adopted from reference [28], copyright 2019, American Chemical Society.

The poor shape stability of PCMs has limited their applications in thermal energy storage. However, to increase the capacity for thermal energy storage, the hybrid composites were developed using microcapsule coating, polymer shaping techniques, and porous support material adsorption technology. Regarding hybrid composites, transparent wood combined with a mixture of polymer and PEG/polymethyl methacrylate (PMMA) can be used for thermal energy storage [28,39,40,41]. To prepare a composite denoted as transparent wood for thermal energy storage, silver birch wood was delignified, loaded with highly miscible PEG 70 wt%, PMMA 30 wt%, and 2,2′-azobis(2-methylpropionitrile) (AIBN) was used as an initiator. Molten PEG-1000 was used for the preparation of the composite and the overall temperature was marinated at 45–70 °C during the preparation process. The PEG/PMMA mixture interacted well with the wood cell wall, according to the scanning electron microscope images (Figure 5a). This can be rationalized by good miscibility of the hydrophilic PEG phase with the wood cell wall. Chemical interactions between the wood composites showed intermolecular interactions between the wood, PEG and PMMA, effectively preventing the leakage.

Thermal storage cycles show that the latent heat of melting and crystallization during thermal storage cycles are approximately 76 and 74 J g^−1^, respectively. When the melting temperature reaches the molten state of PEG, it stores latent heat, which is then released once PEG crystallizes. However, the PEG maintains its melting and crystallization behavior because of the phase change phenomenon of PEG/PMMA, which is very important to thermal performance. Consequently, the transparent wood for thermal energy storage shows excellent thermal stability after 20 mm thermal cycles. Overall, the TGA analysis showed that in the absence of oxygen, it is thermally stable below ~290 °C and, in an oxidative environment, can stay below ~190 °C (Figure 5b). It only showed a weight loss of 1.0%, attributed to residual moisture evaporation.

Similarly, transparent wood for thermal energy storage exhibits switchable optical transmittance properties, like the optical transmittance, which increases by lowering the thickness as the melting phase arrives by 84%, while, as the thickness of the sample increases during crystallization, the optical transmittance is reduced to 68% (Figure 5b). At the same time, transparent wood for thermal energy storage showed excellent mechanical properties, i.e., the prevention of fracture of transparent wood for thermal energy storage is about ten times higher than that of glass. That proves to be the most suitable candidate for heat storage and light transmittance for energy saving in buildings and towers with thermal insulation properties.

**Figure 6:**
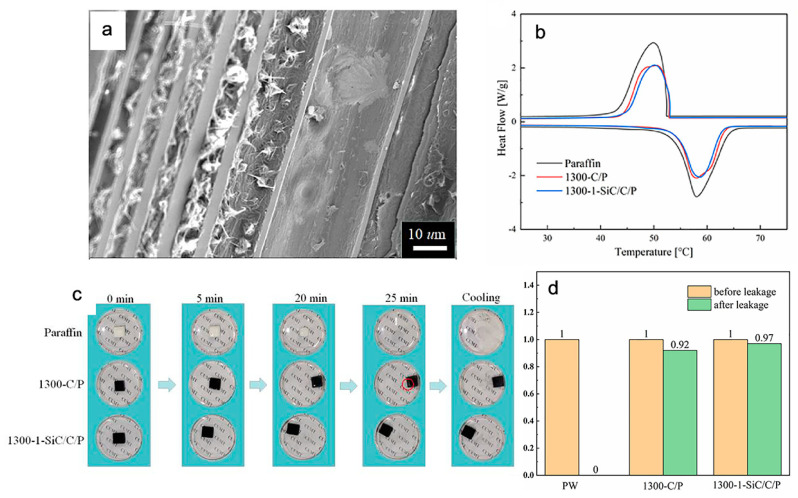
(**a**) SEM micrometer image of SiC/PCCs displaying filling of PW in the microchannels of the carbon skeleton; (**b**) differential scanning calorimetric curves of PW and two different samples of PCC; (**c**) photographs of leakage tests of PW and PCCs upon heating at 80 °C, red circle showing a slight leakage of the sample and (**d**) pictorial representation of weight loss of PCC samples before and after conducting leakage test. Note: SiC = silicon carbide, PW = paraffin wax, PCCs = phase change composites. Adopted with permission from reference [42], copyright 2022, Elsevier B.V.

Rao et el. synthesized phase change composites by using pine wood as a supporting matrix, paraffin wax as PCM and silicon carbide nanowires to enhance thermal conductivity as well as inhibit leakage (Figure 6) [42]. A vacuum immersion process was used to fill paraffin wax into the carbon skeleton microchannels, and silicon carbide nanowires were firmly embedded with paraffin wax as fillers in the channels. This created a two-stage network structure for the adsorption of PCM to form phase change composites names 1300-C/P and 1300-1-SiC/C/P (Figure 6a). The sample paraffin wax and the phase change composites have similar differential scanning calorimeter curves, which indicates that the samples do not react chemically, when immersed in the carbon skeleton (Figure 6b).

The phase change composites also showed the same phase change enthalpies as the paraffin wax, which were 204.7 J g^−1^ during melting and 204.3 J g^−1^ during the solidification process, on the differential scanning calorimeter curves during melting and freezing processes. Due to the inclusion of thermal filler in the composites, enthalpies of phase change composites (128.4 J g^−1^ during melting and 128.3 J g^−1^ during solidification) become lower than the paraffin wax. By heating phase change composites samples in a high-temperature furnace that is hotter than their melting point, leakage test results were compared with paraffin wax. A small amount of leakage was seen on the sample surface after 25 min of heating at 80 °C. This was primarily caused by inability of microchannels to hold paraffin wax due to insufficient capillary suction. In contrast, the silicon carbide/wood carbon skeleton-supported porous structure with paraffin wax can maintain its shape well without apparent leakage (Figure 6c). Silicon carbide nanowires can improve phase change composites ability to stop paraffin wax from leaking out of them by creating stronger suction. Samples 1300-1-SiC/C/P lost only 3 wt% of their original mass following a leakage test, whereas samples 1300-C/P lost 8 wt% (Figure 6d). Herein, thermal conductivity significantly increased for phase change composites (0.78 W m^−1^ K^−1^) as compared to the original paraffin wax (0.2 W m^−1^ K^−1^).

**Figure 7:**
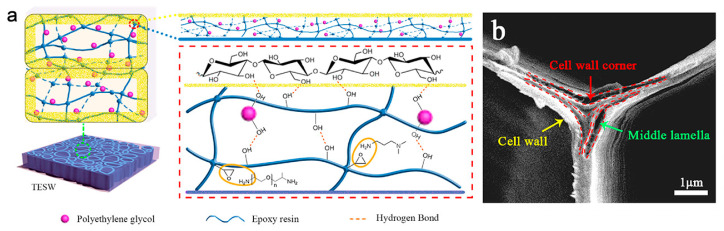
(**a**) Illustration of hydrogen bonding mechanism in TESW and (**b**) cross-sectional image of DW. TESW = transmittance energy storage wood. Reproduced with permission from reference [43], copyright 2021, Elsevier B.V.

Likewise, Guo et al. prepared transmittance energy storage wood using balsa wood as a supporting material to encapsulate PEG and epoxy resins (Figure 7) [43]. The chemical interactions were verified and demonstrated that EP and PEG were merged by a hydrogen bond to form a 3D-network structure (Figure 7a). The formation of a hydrogen bond can strengthen the bonding between the epoxy resin and PEG because it is a type of intermolecular force stronger than van der Waals forces. The PEG/epoxy resins absorption peak at 942 cm^−1^, which is attributed to the -CH_2_ vibration in the transmittance energy storage wood spectrum, shows an intermolecular interaction between the PEG/epoxy resins PCM and the delignified wood model. Resultantly, encapsulation is facilitated by the ability of the cellulose molecular chain in delignified wood to form hydrogen bonds with the PEG/epoxy resins PCM molecular chains. After treatment, epoxy resin can incorporate a 3D cross-linking network structure, which increases the mechanical strength of delignified wood and forms hydrogen bonds with PEG to prevent PEG from leakage. Finally, the benefits of the stability of thermal energy storage and anti-leakage performance of transmittance energy storage wood composites were obtained.

Due to the removal of lignin, cracks appear in the triangular region where three cells intersected; however, the middle lamella separated, which facilitated the infiltration of PEG/epoxy resins PCM (Figure 7b). As a result, the PEG had good fluidity and compatibility with wood after delignification. After infiltration, the microstructure pattern of wood was preserved, showing that the PEG/epoxy resin PCM can partially replace the lignin site and improve the cytoskeleton. There were no observable interfacial gaps between PEG/epoxy resins PCM and the cell wall of the wood. Additionally, epoxy resins as a carrier coated and fixed with PEG can resolve PEG leakage. They can also control the free movement of PEG during PEG crystallization or melting by the 3D cross-linking network structure of epoxy resins.

Also, the enthalpy values of transmittance energy storage wood composites and PEG/epoxy resins PCMs relate to the proportions of PEG contents. Such as, the highest phase-change enthalpy value and best thermal properties were found in transmittance energy storage wood composites with an 80 wt% PEG content. For instance, high latent heat values during energy storage and release were obtained as 134.1 and 122.9 J g^−1^, respectively. In the overall process, transmittance energy storage wood composites contain molten PEG enclosed in the delignified wood template and surrounded by epoxy resin when the temperature is higher than the melting temperature, at which point, the PEG melts and the transmittance energy storage wood stores latent heat. On the other hand, the PEG crystallizes and the transmittance energy storage wood releases the latent heat when the temperature drops to the crystallization temperature.

**Figure 8:**
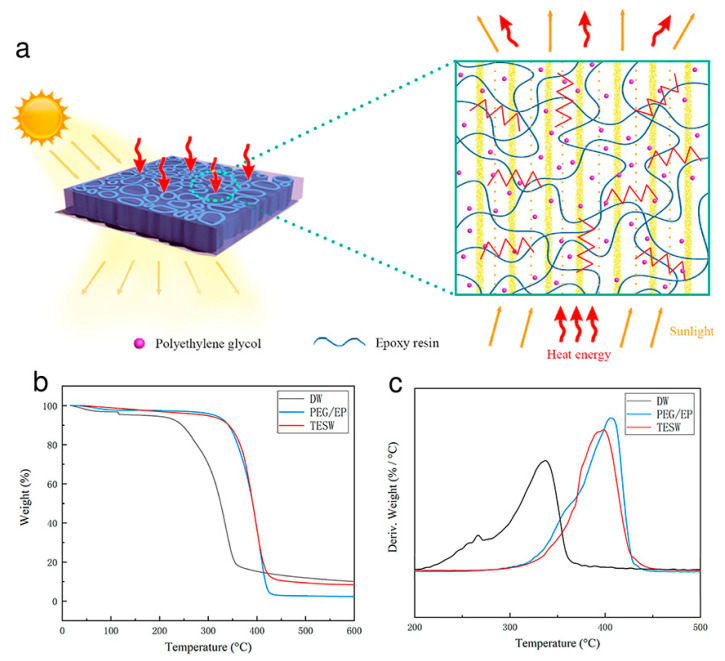
(**a**) Pictorial representation of optical transmission and heat conduction mechanism occurring in TESW composite; (**b**) thermogravimetric analysis of TESW composite and reference DW, PEG/EP PCM and (**c**) DTA curves of TESW composites and reference compounds. Abbreviations in the figures are as follows: DW = delignified wood, PEG = polyethylene glycol, EP = epoxy resin and TESW = transmittance energy storage wood. Reproduced with permission from reference [43], copyright 2021, Elsevier B.V.

Transmittance energy storage wood has a low thermal conductivity of 0.22 W m^−1^ K^−1^, which results in effective thermal insulation performance (transmittance up to 80.89%). That efficiently reduced heat exchange between the inside and outside of the building low thermal conductivity facilitates the overall process. The diffusion-controlled vibrations of the polymer matrix molecular chains, the lattice phonons, and the filled lattice phonons interact to cause heat transfer in transmittance energy storage wood composites (Figure 8a). PEG/epoxy resins PCM and transmittance energy storage wood composites demonstrated good thermal stability with almost no weightlessness in the temperature range of 30–290 °C. PEG/epoxy resins PCM and transmittance energy storage wood composites, however, lost their weight as the temperature was raised (to about 300 °C). At 355 °C, 405 °C, and 399 °C, the maximum weight loss rates of delignified wood, PEG/epoxy resins PCM and transmittance energy storage wood, respectively, were noted (Figure 8b,c).

Indicating that no thermal weight loss occurred during the phase change and that the composite did not volatilize or decompose, the thermogravimetric analysis curves of PEG/epoxy resins PCM and transmittance energy storage wood showed straight lines from room temperature to a temperature above the phase-change temperature. Thus, transmittance energy storage wood proved to be a special kind of transparent building material, which has good thermal insulation properties that can benefit the environment by reducing energy consumption, as well as the economy, by increasing indoor thermal comfort and reducing the need for cooling and heating. The thermal insulation and good heat storage capabilities of transmittance energy storage wood are combined. While reducing the amount of energy used for daytime lighting, transmittance energy storage wood can efficiently direct sunlight into the home and provide even, consistent illumination throughout the day. The physicochemical parameters of the PCMs discussed in this section are compiled in Table 2.

## 4. Carbonized Wood-Based Form-Stable PCMs

Biomass-based materials i.e., eggplant, winter melon, pumpkin, cotton, potatoes and succulents have been carbonized to deal with the issue of PCMs seepage and low thermal conductivity and reported as precursors for the formation of stabilized PCMs [44,45,46,47,48]. These biomass-based porous carbon skeletons exhibit capillary forces and pore-locking action that can efficiently avoid the weight loss of PCMs during the phase transition process. It is unknown, though, whether the capillary force and carbonization structures are enough to form long-duration form stable PCMs composites. In this regard, wood as a naturally porous material is commonly characterized for its sustainable, renewable and environmentally friendly properties. Therefore, wood-derived materials were employed as the base of a supporting framework for constructing form stable PCMs [49,50].

Previously, some methods for improving the thermal conductivity of supporting frameworks through high-temperature carbonization have been published. Since the phonon movement is made easier through a high degree of graphitization, this facilitates heat exchange [51]. Carbonized wood can not only adopt the cell structure of the original wood for packing the PCMs, but it also contributes to the thermal conduction and light absorption of the composite PCMs [52]. Notable here, the problems of carbonized wood-based composite PCMs on form stability and photothermal transformation capacity are less explored.

**Figure 9:**
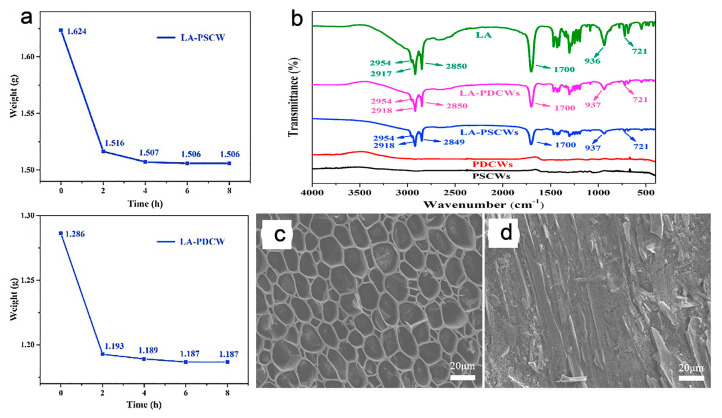
(**a**) Weight-loss graphs of LA/PCWs (time vs. weight) data was taken at 60 °C for 8 h; (**b**) FT-IR spectra of references and PCWs; (**c**) the SEM image of LA showing the complete packaging effect into the PCWs matrix and (**d**) SEM image of LA/PCWs shape-stable composite PCWs. Abbreviations in the figures are as follows: LA = lauric acid, PCW = porous carbonized wood. Adopted with permission [53], copyright 2019, Elsevier B.V.

Li et el. synthesized the porous structure of sycamore wood after delignification and carbonization treatments as encapsulation scaffolds for lauric acid, used as a PCM, for the synthesis of the shape-stable composite of porous carbonized wood (PCWs) (Figure 9) [53]. Lauric acid is the most widely used organic PCM that can easily mix and stay in the pores of absorbent materials such as wood through surface tension and capillary force. The carbonization of bleached wood has the potential to reduce the heat loss during phase transition process and apparently decrease the limit of matrix on phase change behavior of PCMs, both of which are beneficial to keep the heat storage capacity. The leakage from surface due to excessive surface adsorption and small traces of leakage from macropores of shape-stable composite PCWs were observed. The encapsulation mass ratios of shape-stable composite of PCWs were 80.6 wt% and 81.1 wt% respectively, showing that the weight of composite almost remained unchanged after 8 h (Figure 9a). It demonstrated that the shape-stable composite PCWs had excellent form stability, which may have resulted from capillary force and surface tension. PCMs would not leak, even in their molten state, because of the restrictive effect, which guaranteed the stability of their shape. Their Fourier transform infrared spectra also point toward the stable package performance of lauric acid, because the peaks of lauric acid and shape-stable composite PCWs are closely matched (Figure 9b). That is mainly due to the capillary force and surface physisorption of PCWs without chemical influence, further demonstrating that the lauric acid was securely filled in PCWs. The extensive surface area was obtained due to high carbonization ratio that served as adsorption sites, and a lot of pore space served as packaging sites for lauric acid.

The scanning electron microscopic images of shape-stable composite PCWs showed that the surface adsorption of lauric acid was not strong enough, and the weak compatibility with lauric acid (Figure 9c). Despite some traces of lauric acid being separated from the walls, since the walls were closed, it had no impact on the substrate’s overall package performance. Consequently, the PCWs overall exhibited a high level of packaging capacity. In addition, the shape-stable composite PCMs showed high thermal enthalpy of 178.2 J g^−1^, illustrating outstanding package performance and excellent thermal stability in a low-temperature environment. At the same time, the thermal conductivity was relatively lower (0.270 W m^−1^ K^−1^) compared to lauric acid, possibly due to insufficient encapsulation of trace macropores, which slowed down the heat transfer rate and resultantly reduced the overall thermal conductivity. Furthermore, it was found that the carbonization treatment had little effect on increasing thermal conductivity, which could be the result of incomplete amorphous carbon graphitization. In conclusion, PCWs as packing matrices can significantly increase the heat storage capacity because of their high porosity and large pore volume.

**Figure 10:**
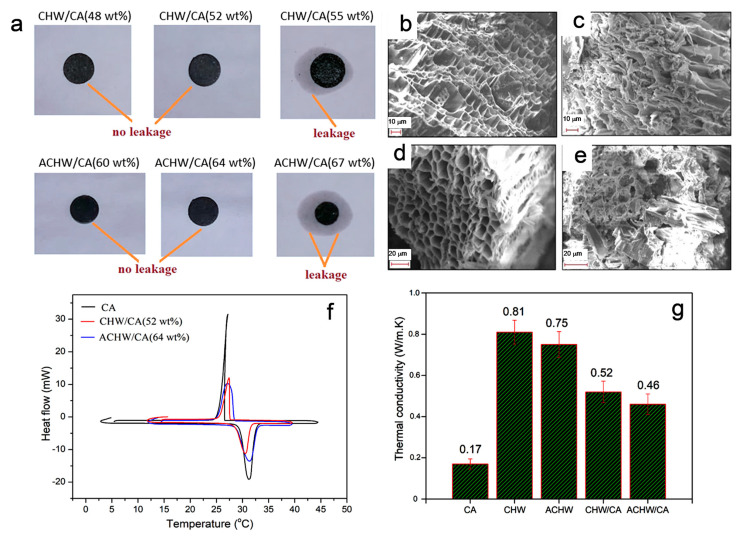
(**a**) Photos of leakage tests of composite PCMs (CHW/CA and ACHW/CA); (**b**–**e**) SEM microstructures of CHW, seepage-free composites CHW/CA, ACHW/CA; (**f**) DSC thermograms of reference (pure CA) and composites and (**g**) bar graph of thermal conductivity trends of reference CA, CHW, ACHW and leakage-free PCM composites. Abbreviations in the figures are as follows: CA = capric acid, ACHW = activated carbonized hazelnut wood, CHW = carbonized hazelnut wood. Reproduced with permission from reference [54], copyright, 2021, Wiley VCH.

Hazelnut wood was used as an inexpensive material to increase the thermal energy transfer rate of PCM with a porous carbon skeleton by Saleh et al. (Figure 10) [54]. Hazelnut wood is a suitable prospective source because factories that process hazelnut can produce them at a low-cost and in large amounts. Hazelnut wood wastes were tested to determine if they could be used to make porous carbon materials and utilized as a supportive substance in forming leakage-free PCMs composite for energy management in buildings. They produced carbonized hazelnut wood and chemically activated carbonized hazelnut wood as supportive media for the shape stabilization of capric acid. Due to its appropriate melting-solidification temperature (~29.5 °C) and comparatively significant latent heat thermal energy storage capacity in the range of 156.4~215.7 J g^−1^ [54,55,56], the capric acid was chosen as the PCM for the use of thermoregulation in building envelopes. Through vacuum impregnation, leakage-free activated carbonized hazelnut wood/capric acid and carbonized hazelnut wood/capric acid composites were synthesized, and the filter paper test was performed to check the leakage of the prepared samples (Figure 10a). The final holding capacity of activated carbonized hazelnut wood/capric acid and carbonized hazelnut wood/capric acid composites was 40–70%, after heating at the liquefaction temperature of capric acid for 30 min.

In conclusion, the activated carbonized hazelnut wood has a higher rate of capric acid adsorption than that of carbonized hazelnut wood, as determined by seepage tests. However, the capric acid levels captivated by both samples demonstrate porous architectures that are ideal for PCM impregnation. Since the successful forces of surface tension and capillary action between carbonized hazelnut wood/activated carbonized hazelnut wood and capric acid, capric acid adsorption occurred uniformly throughout the porous designs of carbonized hazelnut wood and activated carbonized hazelnut wood (Figure 10b–e). As a result, the porous skeleton of carbonized hazelnut wood and activated carbonized hazelnut wood offers mechanical advantages and prevents capric acid from leakage. The DSC results confirmed the phase transition temperatures of activated carbonized hazelnut wood/capric acid and carbonized hazelnut wood/capric acid composites, which relatively differ from the pure form of capric acid (Figure 10f). This change is because the composites hold in porous structures by capillary and surface tension forces. Other than that, they got higher latent heat thermal energy storage capacities for activated carbonized hazelnut wood/capric acid and carbonized hazelnut wood/capric acid composites (111.3–110.3 J g^−1^ and 136.7−135.9 J g^−1^), respectively.

Consequently, the phase change enthalpies of the carbonized hazelnut wood/capric acid and activated carbonized hazelnut wood/capric acid composites are improved. They also found high thermal conductivities for prepared samples compared to pure capric acid, i.e., pure capric acid (0.17 W m^−1^ K^−1^), carbonized hazelnut wood (0.81 W m^−1^ K^−1^) and activated carbonized hazelnut wood (0.75 W m^−1^ K^−1^) (Figure 10g). Carbonized hazelnut wood has relatively higher conductivity because of its two folds larger pore volume than activated carbonized hazelnut wood. As activated carbonized hazelnut wood possesses open pores that are much more permeable than the closed pores of capric acid, which contain molecules with low thermal conductivity (about 0.023–0.026 W m^−1^ K^−1^) compared to those of carbonized hazelnut wood and capric acid samples. This difference may be due to the greater impregnation of capric acid by activated carbonized hazelnut wood and because of lower thermal conductivity of activated carbonized hazelnut wood. To sum up the study, leakage-free composite PCMs can be cost-effective and eco-friendly new types of fillers for efficient storage purposes, as well as for thermal management in concrete and plaster buildings.

**Figure 11:**
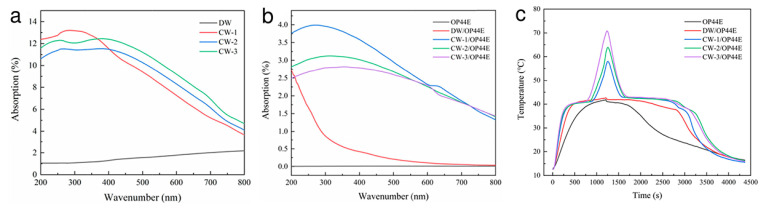
(**a**,**b**) The absorption graphs of carbonized wood samples, DW/OP44E and CW-OP44E and (**c**) photothermal behavior of the samples. DW stands for delignified wood and CW stands for carbonized wood and OP44E is supporting paraffin material. Reproduced with permission from reference [57], copyright 2021, Elsevier B.V.

The effective utilization of solar energy is challenging; however, photothermal or photochemical conversion by utilizing the PCMs can provide a better use of solar energy for household usage. In this regard, a photothermal conversion method is the most efficient technique for the utilization of solar energy. Concerning instantaneous solar rays that could generate high-energy photons, the utilization of suitable PCMs helps to overcome this problem by maintaining the storage of thermal energy. Recently, carbonized balsa wood with regular pore sizes was used in PCMs for photothermal conversion using a vacuum impregnation method (Figure 11) [57]. This method effectively maintained the thermal properties and microporous structure of wood as well as for the newly constructed composite PCMs, which were verified by several spectroscopic methods, i.e., Fourier transform infrared spectroscopy, differential scanning calorimetry and thermogravimetric analysis and scanning electron microscope images, etc.

Interestingly, the phase change temperature and latent heat of carbonized wood-3/OP44E material were significantly improved and measured as 41 °C and 206.3 J g^−1^, respectively, note that the PCMs were packed in a porous structure of carbonized wood with OP44E (OP44E is a member of paraffin family). The ability of carbonized wood to absorb light, especially in the range of (400–800 nm), is significantly greater than that of native delignified wood. This is because of the excellent photo-thermal activity of carbon materials, which includes superior light absorption and heat conduction ability (Figure 11a). Similarly, OP44E has almost no ability to absorb light. As a result, when OP44E was enclosed in carbonized wood, the light absorption ability of the carbonized wood/OP44E sample significantly increased (Figure 11b). Moreover, the photothermal conversion performance of composite PCMs was evaluated under a bright xenon lamp. The graph plot between time and temperature showed that upon light illumination, the temperature of delignified wood/OP44E and carbonized wood/OP44E samples raised sharply, in contrast with the OP44E, which heated slowly (note that the OP44E is supporting paraffin material). This demonstrates unambiguously that OP44E has poorer light absorption properties than delignified wood and carbonized wood samples (Figure 11c). Carbonized wood/OP44E heats up more quickly than delignified wood/OP44E, which is consistent with the light absorbing ability of the materials.

Three different carbonized wood composites achieved maximum photothermal temperatures of 70 °C, 64 °C and 58 °C after 1240 s time lapse for carbonized wood-3/OP44E, carbonized wood-2/OP44E, and carbonized wood-1/OP44E samples, respectively. The temperature only rises to 42 °C for delignified wood/OP44E, slightly higher than that of OP44E, indicating that the two materials transfer heat at various rates. More specifically, the composites made of carbonized wood and OP44E effectively increase the capacity of material for photothermal conversion, and when compared to native OP44E, the temperature of the carbonized wood-3/OP44E material rises by a significant number of 28 °C. The photothermal conversion efficiency of the composite PCMs reached 90%, leading to the conclusion that they have a suitable photothermal conversion property. It is thus appropriate for potential use in thermal energy storage applications.

**Figure 12:**
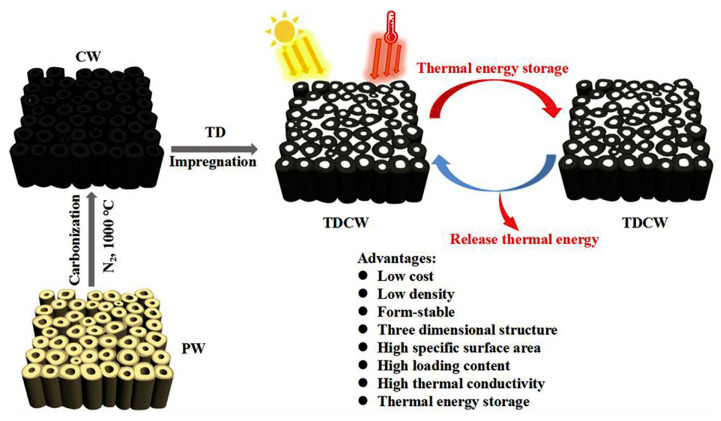
Diagram showing the carbonization of wood/impregnation of TD mechanism and formation of TDCW. The advantages of this strategy and thermal energy storage/release cycle are also mentioned. TD = 1-tetradecanol, TDCW is PCM composite made of 1-tetradecanol and carbonized wood. Adopted after permission [52], copyright 2018, Elsevier BV.

A novel material for the support of PCM was demonstrated by a 3D porous wood structure because of its large surface area, low cost and excellent thermal conductivity. Meanwhile, wood has a low density and, especially after carbonization, it exhibited a declined density of about 0.63 g/cm^3^. Previously, carbonized wood-based composite PCMs were developed with low density, low cost, 3D structures, high loading content and high thermal conductivity (Figure 12) [52]. Poplar wood was used as a supportive structure to load 1-tetradeconal to synthesize form-stable phase change material. The composite is based on carbonized wood for the impregnation of 1-tetradeconal. The chemical compatibility observed by X-ray diffraction and Fourier transform infrared spectra of the composite material, showed excellent graphitic crystalline structure of carbonized wood fully loaded with 1-tetradeconal and did not affect each other. They achieved maximum surface area and pore volume of 235.37 m^2^/g (60 times greater than pristine wood) and 0.13 cm^3^/g (30 times higher than pristine wood), respectively.

The adsorption and desorption curves of carbonized wood, on the other hand, revealed that it has mesoporous and microporous structures, and that it can confine 1-tetradeconal without leakage. After carbonization, the dimensions of the wood decreased, but it proved to be a beneficial porous structure for the impregnation of 1-tetradeconal into carbonized wood, and it also has the advantage of leakage prevention of the melted 1-tetradeconal. This 1-tetradeconal-based carbonized wood PCM material showed a high latent heat of storage of 165.8 J g^−1^ and thermal reliability after testing by 200 heating/cooling cycles, which is also suitable for below 120 °C temperature. In this way, this material is favorable for floor, building wallboards, food and medical storage, as well as in other fields for thermal energy storage at low-temperature management. Moreover, this novel PCM material exhibited 114% higher thermal conductivity (0.669 W m^−1^ K^−1^) compared with the 1-tetradeconal precursor (0.312 W m^−1^ K^−1^) at 50 °C, further suggesting its suitability in thermal management systems. The key physicochemical parameters of the PCMs materials discussed in this section are summarized in Table 3. 

## 5. Concluding Remarks and Outlook

In summary, the recent developments for building form-stabilized PCMs consisting of biomass-based media as a supporting material are discussed in this review. We especially emphasized wood, either the hybrid or polymers wood-based material or wood composites, because the recyclable nature of wood has the advantage of low PCM preparation cost. Moreover, the different uses of wood-based form-stabilized PCMs were demonstrated for anti-leakage benefits as well as for improved thermal conductivity. The PCMs design rationale, their composition and the obtained benefits after amplifications are discussed. We briefly discussed the encapsulation methods of PCMs and the delignification of wood. The wood-based PCMs improved heat transfer performance and minimized heat loss. However, there are significant issues with the PCMs adaptability and ease of handling that still need to be resolved. For instance, the introduction of flame retardants into composite PCMs will help to reduce the overall loading needs and enhance the latent heat capacity. Additionally, holding high thermal conductivity and being thermally stable, PCM materials should be developed using superficial and efficient preparation routes as support for form-stabilized PCMs composites in various thermal energy storage systems with variable temperature ranges. In the current scenario, where sustainability and energy efficiency are at the forefront of the research, we think that organic PCMs will play an increasingly significant role. The studies on enhancing their properties, i.e., high thermal conductivity, leakage-free and flame-retardant nature, will be the key cornerstone that allows for the practical usage of these PCMs. All this information is useful for in-depth understanding and improving energy conversion efficiency from the mechanism and developing new PCMs suitable for applications.

## Figures and Tables

**Table 1:** Physicochemical parameters of selected PCM materials mentioned in this section.

PCM	Supporting Material	Preparation Method	Leakage Test Time /Temperature	Thermal Conductivity (W/m K) /Stability/Latent Heat (J g^−1^)	Ref.
PEG-1000/WF	Wood flour	Vacuum distillation	1 h/65 °C	-/stable up to 400 °C/108.6	[34]
PEG/FDU-15	Mesoporous carbon (FDU-15)	Melting impregnation	0.5 h/80 °C	0.44/high stability/81.7	[35]
MicroPCM/ graphene	Balsa wood	Vacuum impregnation	-	0.873/20~300 °C/44.3	[36]
PEG-800/graphene aerogel	Poplar wood	Hydrothermal	0.5 h/60 °C	0.374/20~600 °C/19.8	[37]
PEG-1500/delignified balsa wood	Balsa wood	Vacuum impregnation	8 h/80 °C	-/good stability/134.0	[38]

**Table 2:** Physicochemical parameters of representative PCM materials of this section.

PCM	Supporting Material	Preparation Method	Leakage Test Time /Temperature	Thermal Conductivity (W/m K) /Stability/Latent Heat (J g^−1^)	Ref.
PEG-1000/PMMA	Delignified silver birch wood	Vacuum impregnation	-	0.30/~40 °C/76.0	[28]
Paraffin wax/silicon carbide	Pine wood	Vacuum impregnation	25 mint/80 °C	0.78/~51 °C/128.4	[42]
PEG-800/epoxy resins	Balsa wood	Vacuum impregnation	-	0.22/30~290 °C/134.1~	[43]

**Table 3:** Physicochemical parameters of selected PCM materials discussed in this section.

PCM	Supporting Material	Preparation Method	Leakage Test Time /Temperature	Thermal Conductivity (W/m K) /Stability/Latent Heat (J g^−1^)	Ref.
Lauric acid/ carbonized wood	delignified sycamore wood	vacuum impregnation	8 h/60 °C	0.270/100~300 °C/178.2	[53]
Capric acid/ hazelnut wood	Carbonized hazelnut wood	vacuum impregnation	1 h/45 °C	0.81/>160 °C/111.3~135.9	[54]
Carbonized wood/OP44E	OP44E paraffin	vacuum impregnation	-	0.182/160~310 °C/206.3	[57]
1-tetradeconal/carbonized wood	Carbonized poplar wood	vacuum impregnation	-	0.669/120~200 °C/138.7~165.8	[52]

## Data Availability

Not applicable.
